# Sophisticated Structural Tuning of NiMoO_4_@MnCo_2_O_4_ Nanomaterials for High Performance Hybrid Capacitors

**DOI:** 10.3390/nano12101674

**Published:** 2022-05-14

**Authors:** Yifei Di, Jun Xiang, Nan Bu, Sroeurb Loy, Wenduo Yang, Rongda Zhao, Fufa Wu, Xiaobang Sun, Zhihui Wu

**Affiliations:** 1 School of Materials Science and Engineering, Liaoning University of Technology, Jinzhou 121001, China; diyifei97@126.com (Y.D.); bn1376980216@163.com (N.B.); loysroeurb999@gmail.com (S.L.); 18342841189@163.com (W.Y.); lilac_rain@126.com (X.S.); 2 Liaoning Brother Electronics Technology Co., Chaoyang 122000, China; fyzl86@126.com

**Keywords:** supercapacitors, NiMoO_4_@MnCo_2_O_4_, microstructure, electrochemical performance, cycling stability

## Abstract

NiMoO_4_ is an excellent candidate for supercapacitor electrodes, but poor cycle life, low electrical conductivity, and small practical capacitance limit its further development. Therefore, in this paper, we fabricate NiMoO_4_@MnCo_2_O_4_ composites based on a two-step hydrothermal method. As a supercapacitor electrode, the sample can reach 3000 mF/cm^2^ at 1 mA/cm^2^. The asymmetric supercapacitor (ASC), NiMoO_4_@MnCo_2_O_4_//AC, can be constructed with activated carbon (AC) as the negative electrode, the device can reach a maximum energy density of 90.89 mWh/cm^3^ at a power density of 3726.7 mW/cm^3^ and the capacitance retention can achieve 78.4% after 10,000 cycles.

## 1. Introduction

With the development of the world economy, environmental pollution is caused by the excessive burning of traditional fossil fuels, which poses a serious threat to the goal of human sustainable development [1]. Supercapacitors (SCs), as a new environmentally friendly electrochemical energy storage device, have attracted extensive attention from researchers. The selection of electrode material is an important factor for energy storage performance. Developing an electrode material with excellent electrochemical performance has become key to the future development of SCs [2,3,4,5]. Transition metal oxides possess high specific capacitance, superior cycling performance and abundant valence states, such as NiMoO_4_, MnCo_2_O_4_, NiCo_2_O_4_ and ZnCo_2_O_4_. They have been widely reported due to their large theoretical capacitance, excellent redox performance and environmental friendliness [6,7,8,9,10].

NiMoO_4_ is a very suitable electrode material for SCs because of its advantages of better electrochemical performance and low price [11,12,13]. However, there are still many problems such as low theoretical utilization value, poor cycle life and low conversion performance at a higher rate [14]. Xuan [15] et al. prepared a NiMoO_4_@Co_3_O_4_ composite nanoarray electrode. The pseudocapacitance performance of the prepared NiMoO_4_@Co_3_O_4_-5H composite was 1722.3 F/g at the current density of 1 A/g, and the capacitance retention rate of 91% was realized by the 6000 cycles test. Feng [16] et al. prepared hierarchical flower-like NiMoO_4_@Ni_3_S_2_ composite material on a 3D nickel foam matrix by the hydrothermal method. The specific capacity was 870 C/g at 0.6 A/g, and the capacity retention rate was 81.2% after 8000 cycles. Transition metal oxide MnCo_2_O_4_ with excellent electrochemical performance is very suitable for the electrode material of SCs, because its Mn ion can offer high electron conductivity and excellent rate performance, and cobalt ion has high oxidation potential. However, they can also demonstrate poor application, such as poor cycling performance, poor electrical conductivity and so on, which greatly affect the practical application of SCs [17,18]. Cheng [19] et al. prepared porous MnCo_2_O_4_@NiO nanosheets by hydrothermal synthesis and calcination. The specific capacitance of the electrode material was 508.3 F/g at 2 A/g current density. The 2000 cycles test was applied at 10 A/g current density, and it presented the capacitance retention performance of 89.7%. Liu [20] et al. prepared MnCo_2_O_4_@MnO_2_ nanosheet arrays with core–shell structure on nickel foam by two-step hydrothermal treatment. The surface capacitance of the electrode was 3.39 F/cm^2^ at a current density of 3 mA/cm^2^. Furthermore, the capacity retention rate was 92.5% by 3000 cycles test at a current density of 15 mA/cm^2^. It could be seen that the composites exhibited excellent electrochemical properties due to their excellent conductivity [21,22,23,24]. It was also confirmed that NiMoO_4_ and MnCo_2_O_4_ have great potential as electrode materials for SCs [25]. The composite electrodes constructed from these two materials can effectively improve the conductivity, specific surface area, and number of reaction sites, thereby improving the overall electrochemical performance. [26,27,28].

In this work, NiMoO_4_@MnCo_2_O_4_ composite electrode material is obtained by the two-step hydrothermal synthesis method. The results show that the NiMoO_4_@MnCo_2_O_4_ electrode has better electrochemical performance than single NiMoO_4_ or MnCo_2_O_4_ electrode, and its electrochemical performance is greatly improved after the composite. At the current density of 1 mA/cm^2^, the specific capacitance of single NiMoO_4_ electrode material is 1656 mF/cm^2^, and the specific capacitance of the single MnCo_2_O_4_ electrode material is 224 mF/cm^2^. Finally, the NiMoO_4_@MnCo_2_O_4_ electrode material is 3000 mF/cm^2^. After 10,000 cycles, the capacity retention rate of NiMoO_4_@MnCo_2_O_4_ electrode material is 96%. NiMoO_4_@MnCo_2_O_4_//AC devices show high electrochemical performance with a maximum energy density of 90.89 mWh/cm^3^ and a power density of 3726.7 mW/cm^3^.

## 2. Experimental Section

### 2.1. Preparation of NiMoO_4_ Nano Pompon-Like Structure Electrode Material

In a typical process, 6 mmol Na_2_MoO_4_·2H_2_O, 6 mmol Ni(NO_3_)_2_·6H_2_O, 1 mmol NH_4_F, and 1 mmol CO(NH_2_)_2_ was added to 50 mL deionized water. After magnetic stirring, the nickel foam was put into the solution and reacted at 120 °C for 12 h, and then it was cleaned by deionized water and anhydrous ethanol to remove surface impurities. The NiMoO_4_ precursor was obtained by drying for 6 h in a drying oven at 60 °C and annealing for 2 h in air at 350 °C.

### 2.2. Preparation of NiMoO_4_@MnCo_2_O_4_ Urchin-like Core-Shell Structure Electrode Material

In a similar process to above, 6 mmol Mn(CH_3_COO)_2_·4H_2_O, 6 mmol Co(NO)_3_·6H_2_O, 5 mmol NH_4_F and 5 mmol CO(NH_2_)_2_ were dissolved in 50 mL deionized water to obtain a homogeneous solution. The nickel foam with NiMoO_4_ was put into this solution, and it kept 140 °C for 8 h. After cooling down to room temperature, the samples were washed, dried, and annealed for 2 h at 350 °C. The mass loading of NiMoO_4_, MnCo_2_O_4_, and NiMoO_4_@MnCo_2_O_4_ is 1.27, 1.02, and 1.91 mg/cm^2^, respectively.

### 2.3. Materials Characterizations

The elemental composition and valence of the samples were characterized by X-ray powder diffraction (XRD, D/max-2500/PC, Rigaku Corporation, Tokyo, Japan) with Cu Kα (λ = 1.5406 Å) and X-ray photo-electron spectroscopy (XPS, ESCALAB250, FEI Company, Waltham, MA, USA). The structure and morphology were investigated by emission scanning electron microscopy (SEM, Sigma500, Zeiss, Jena, Germany), and high-resolution transmission electron microscopy (HRTEM, Tecnai G2 S-Twin F20, FEI Company, Waltham, MA, USA).

### 2.4. Electrochemical Measurements

The electrochemical characteristics of the products were tested by Shanghai CHI660E electrochemical workstation. The sample material was applied as the working electrode, the platinum electrode was utilized as the auxiliary electrode, and Hg/HgO electrode was employed as the reference electrode. The working electrode was processed as a circle with a diameter of 1 cm. Moreover, 3 M KOH solution was used as the electrolyte and the ultrasonic-treated nickel foam was served as the collector. Through cyclic voltammetry (CV), galvanostatic charging–discharging (GCD), electrochemical impedance spectroscopy (EIS) and cycling performance measurements, the electrochemical properties of electrode materials and their application value were analyzed.

Energy density (E) can be obtained from the integral area of discharging curves. Specific capacitance (Cs), power density (P), and coulombic efficiency (η) can be calculated by the following equations:

C_s_ = IΔt_d_/SΔV (1)

P = 3600E/Δt_d_ (2)

η = Δt_d_/Δt_c_ (3)

where I is the current value, Δt_d_ and Δt_c_ represent the discharging time and charging time, S is the geometrical area of the electrode, and ΔV denotes the voltage window.

### 2.5. Fabrication of Asymmetric Supercapacitors

Asymmetric supercapacitors were constructed with NiMoO_4_@MnCo_2_O_4_ as the positive electrode and active carbon as the negative one. The active carbon electrode was made of active carbon, acetylene black, and polyvinylidene fluoride with N-methylpyrrolidone as the solvent in a mass ratio of 7:2:1. The slurry was evenly coated on the nickel foam. The active carbon electrode was vacuum dried for 24 h at 60 °C. The electrolyte of ASCs was PVA-KOH. The preparation process was as follows: 3 g PVA and 3 g KOH were mixed in 30 mL deionized water, and the mixture was heated in an 80 °C water bath for 1 h and stirred continuously until clear.

## 3. Results and Discussion

**Figure 1:**
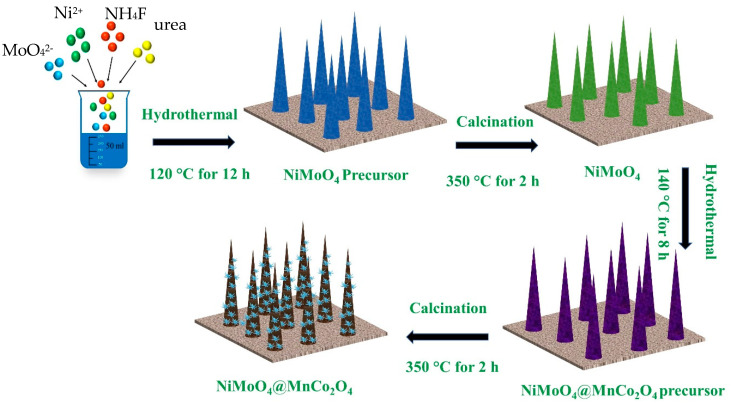
Synthesis schematic of NiMoO_4_@MnCo_2_O_4_ composite electrode.

The NiMoO_4_@MnCo_2_O_4_ composite electrode was synthesized by a two-step hydrothermal method, as shown in Figure 1. Firstly, NiMoO_4_ precursor is grown on nickel foam. Secondly, NiMoO_4_ can be obtained by calcination. Thirdly, the nano needle-like MnCo_2_O_4_ precursor was coated on NiMoO_4_ by the second hydrothermal preparation. Finally, the samples were calcined to obtain NiMoO_4_@MnCo_2_O_4_ on nickel foam.

**Figure 2:**
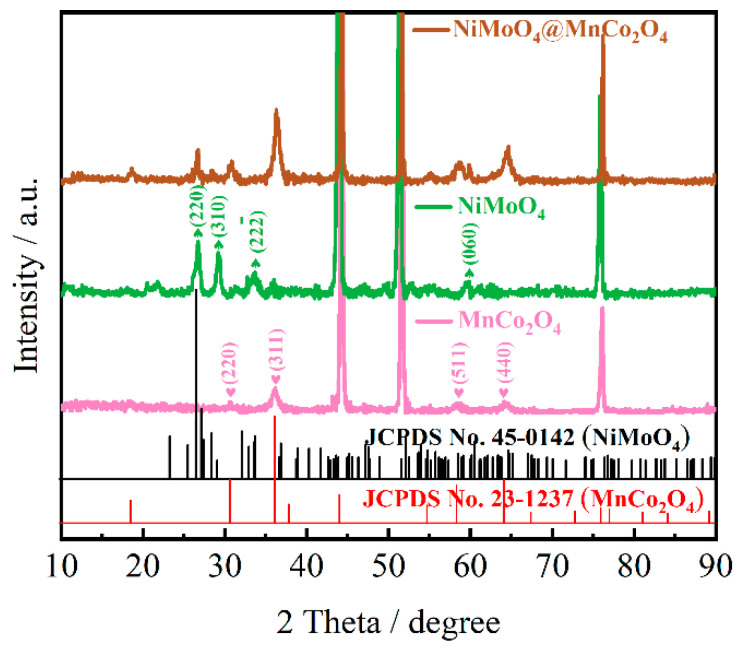
XRD patterns of NiMoO_4_, MnCo_2_O_4_ and NiMoO_4_@MnCo_2_O_4_ electrode materials.

As seen from the XRD results of NiMoO_4_, MnCo_2_O_4_ and NiMoO_4_@MnCo_2_O_4_ electrode materials, it can be observed that the three strong peaks are diffraction peaks of the foamed nickel substrate in Figure 2. When 2θ values are 26.57°, 29.14°, 33.73° and 60.01°, the crystal planes correspond to (220), (310), (22$\bar{2}$) and (060). The crystal structure is consistent with that of NiMoO_4_ (JCPDS No. 45-0142). Meanwhile, the values of 2θ are 30.53°, 35.99°, 57.90° and 63.62° and the diffraction peaks correspond to (220), (311), (511) and (440) crystal planes, which is consistent with the crystal structure of MnCo_2_O_4_ (JCPDS No. 23–1237). Therefore, the diffraction peaks of NiMoO_4_@MnCo_2_O_4_ electrode material prepared under the condition of the best ratio correspond to the diffraction peaks of a single compound.

**Figure 3:**
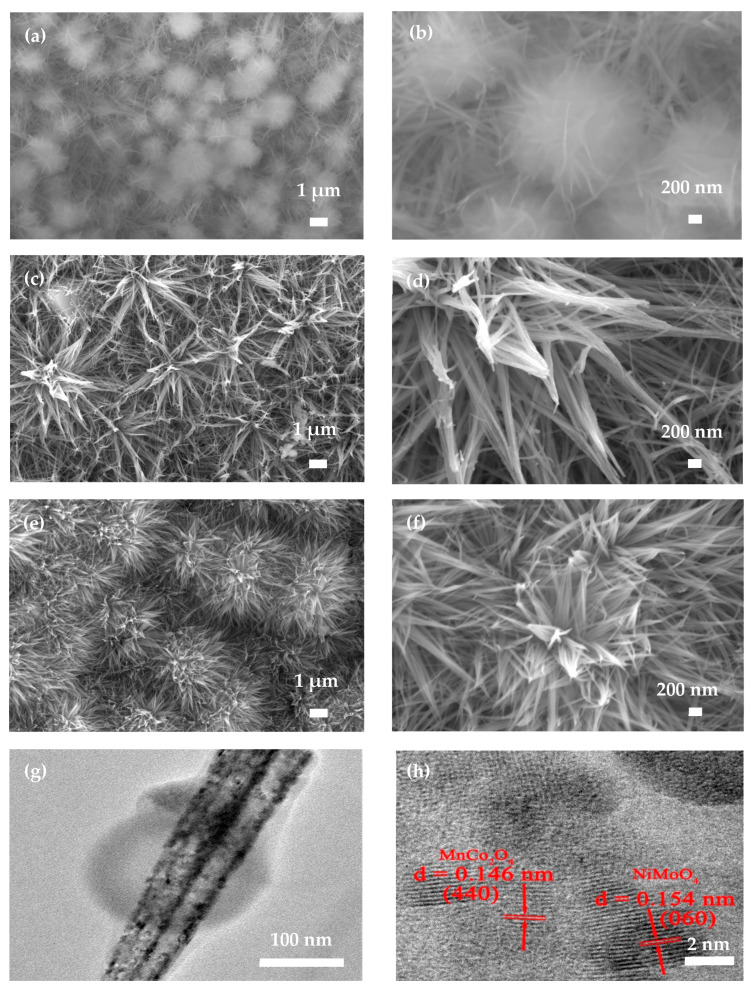
(**a**–**f**) Microstructure of NiMoO_4_, MnCo_2_O_4_ and NiMoO_4_@MnCo_2_O_4_ electrode materials at different multiples; (**g**,**h**) TEM of NiMoO_4_@MnCo_2_O_4_ electrode material.

Figure 3 shows the morphologies of NiMoO_4_, MnCo_2_O_4_ and NiMoO_4_@MnCo_2_O_4_ electrode materials. As seen from Figure 3a,b, the NiMoO_4_ electrode material is nano pompon-like, and there are many intersecting nano needle-like structures densely growing on the nickel foam substrate. As shown in Figure 3c,d, MnCo_2_O_4_ electrode material possesses a nano needle-like structure and uniformly grows on the nickel foam substrate. Figure 3e,f show the micromorphology of NiMoO_4_@MnCo_2_O_4_ electrode material. It can be observed that a large number of uniformly distributed nano needle-like MnCo_2_O_4_ and nano pompon-like NiMoO_4_ grow together to form a uniform and orderly arrangement of nano urchin-like morphology, which increases the specific surface area of NiMoO_4_ electrode and presents a great deal of active sites for rapid transfer between ions and active substances. The gap between the nano needle-like structures allows sufficient Faraday chemical reactions between the active substance and electrolyte, which enhances the electrochemical storage performance. Figure 3g,h show TEM images of NiMoO_4_@MnCo_2_O_4_ electrode material. Figure 3g exhibits the morphology after the composite of NiMoO_4_ and MnCo_2_O_4_. It can be seen from Figure 3h that NiMoO_4_@MnCo_2_O_4_ composite material shows two kinds of lattice fringes; the lattice fringes with the spacing of 0.154 nm correspond to the (060) crystal plane of NiMoO_4_, and the lattice fringes with the spacing of 0.146 nm correspond to the (440) crystal plane of MnCo_2_O_4_. From the stable microstructure of NiMoO_4_@MnCo_2_O_4_, it can be inferred that the composite has multiple ion and electron transport channels and a larger specific surface area, therefore it is beneficial to shorten the ion diffusion path, which makes it advantageous for high storage capacity and rate capacity.

**Figure 4:**
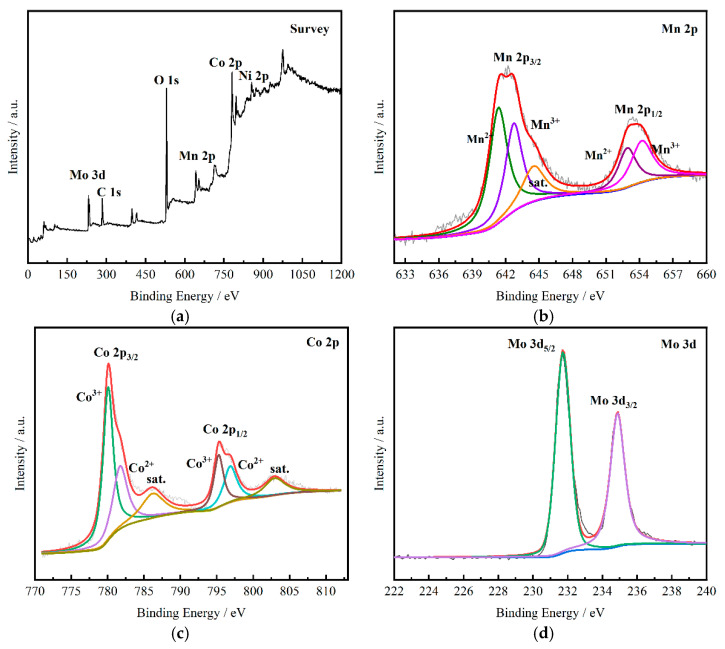

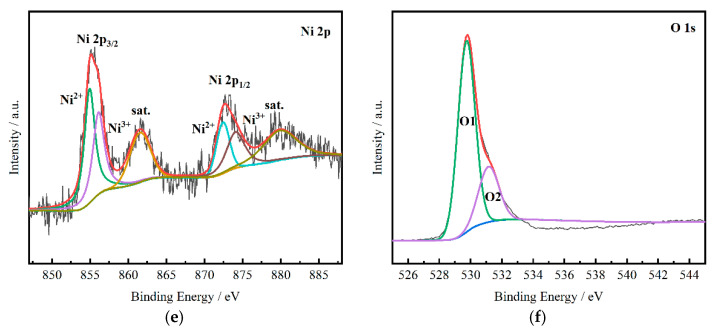
XPS diagram of NiMoO_4_@MnCo_2_O_4_ electrode material: (**a**) Full measurement spectrum; (**b**) Mn 2p; (**c**) Co 2p; (**d**) Mo 3d; (**e**) Ni 2p; (**f**) O 1s.

In order to further investigate the elemental component and different valence states of the prepared NiMoO_4_@MnCo_2_O_4_ composite, XPS tests were carried out on the samples. Figure 4a presents the full measurement scanning spectrum showing the presence of Mn 2p, Co 2p, Mo 3d, Ni 2p, O 1s and C 1s, among which O 1s and C 1s elements are mixed impurities in the test process. In order to identify the detailed valence states of Mn, the high resolution XPS spectrum is present in Figure 4b. The Mn 2p_3/2_ and Mn 2p_1/2_ are found in the two main peaks, respectively, which can be divided into four peaks after fine fitting. The two peaks with a binding energy of 641.4 eV and 652.9 eV can be ascribed to the presence of Mn^2+^. The peaks corresponding to Mn^3+^ are distributed with a binding energy of 644.6 eV and 654.2 eV, respectively. Meanwhile, there is a satellite peak (defined as “Sat.”) at a position with a binding energy of 644.6 eV. According to the Co 2p spectrum of Figure 4c, it was found that two peaks appear at 780 eV and 795.3 eV, corresponding to the two excitation spectra of Co 2p_3/2_ and Co 2p_1/2_. The diffraction peaks corresponding to Co^2+^ have a binding energy of 781.5 eV and 797.3 eV, respectively. The diffraction peaks corresponding to Co^3+^ have a binding energy of 779.9 eV and 795.2 eV, respectively. In Figure 4d, the peaks of Mo 3d spectrum at 231.6 eV and 234.8 eV belong to Mo 3d_5/2_ and Mo 3d_3/2_, respectively. In Figure 4e, Ni 2p spectra can be well fitted into two main peaks, characterized by Ni^2+^ and Ni^3+^ oxidation states. Each peak has its own satellite peak (defined as “Sat.”) at 861.6 eV and 879.9 eV, respectively. Two fitting peaks at 855.1 eV (Ni 2p_3/2_) and 872.9 eV (Ni 2p_1/2_) belong to Ni^2+^, and two fitting peaks at 855.9 eV (Ni 2p_3/2_) and 873.8 eV (Ni 2p_1/2_) belong to Ni^3+^. Figure 4f shows the O 1s region, which can be divided into two peaks (529.8 eV and 531.8 eV). For the binding energy of 529.8 eV, it is attributed to the formation of M-O bond (M=Co, Mn). Therefore, XPS data confirm that the synthesis of NiMoO_4_@MnCo_2_O_4_ is successful [29,30,31].

**Figure 5:**
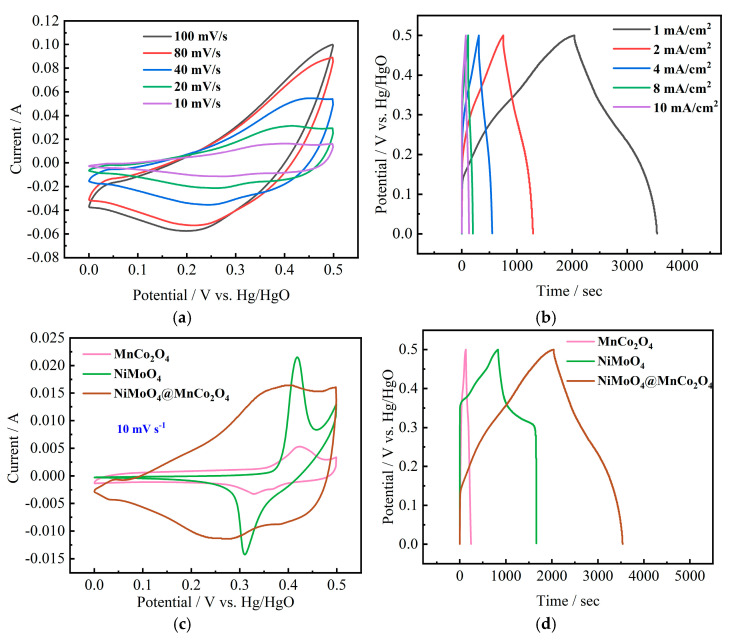

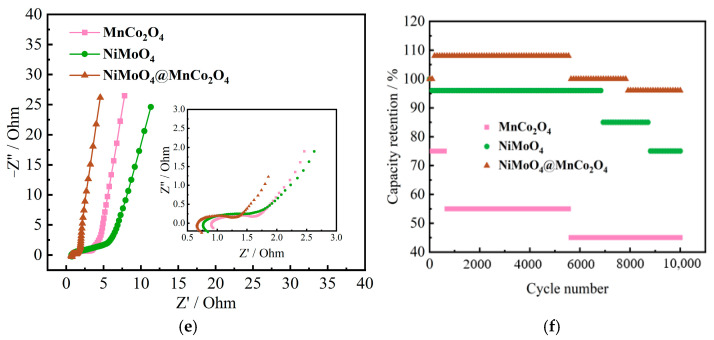
Electrochemical tests of three electrode materials: (**a**) CV curves of NiMoO_4_@MnCo_2_O_4_; (**b**) GCD curves of NiMoO_4_@MnCo_2_O_4_; (**c**) CV curves of three electrode materials; (**d**) GCD curves of the three electrode materials; (**e**) EIS curves of the three electrode materials (inset is the high-frequency region); (**f**) Long cycle curves of the three electrode materials.

Figure 5a shows the cyclic voltammetry (CV) curves of NiMoO_4_@MnCo_2_O_4_ electrode material, which is measured by a scanning rate of 10–100 mV/s and a voltage window of 0–0.5 V, showing excellent rate performance. The visible redox peaks are seen from the curves, indicating that redox reaction occurs in the process of energy storage. Figure 5b presents the galvanostatic charge–discharge (GCD) curves with current density of 1, 2, 4, 8, and 10 mA/cm^2^, the areal capacitance is 3000, 1076, 964, 696, and 580 mF/cm^2^, respectively. The high electrochemical performance is mainly attributed to the nano urchin-like morphology of the material. The nano needle-like structure densely and uniformly distributed on the urchin-like surface provides a larger surface area for electrolyte contact, thus improving the electrochemical performance of the composite.

In order to show the advantages of the composite electrode, NiMoO_4_, MnCo_2_O_4_ and NiMoO_4_@MnCo_2_O_4_ electrode materials are used as working electrodes, respectively, and necessary tests are carried out in a three-system with 3 M KOH solution. Studies have shown that the capacitance of NiMoO_4_ in an alkaline environment is mainly attributed to the reversible redox reaction between the valence states of Ni element, while Mo element does not participate in any reaction, but it helps to improve the conductivity of molybdate. Figure 5c reveals the CV curves of NiMoO_4_, MnCo_2_O_4_ and NiMoO_4_@MnCo_2_O_4_ electrodes at 10 mV/s. Visible redox peaks can be seen from the curves. By comparing the three CV curves, it is obviously observed that the NiMoO_4_@MnCo_2_O_4_ electrode has a larger integral area than NiMoO_4_ and MnCo_2_O_4_ electrode, so it has a larger specific capacitance. These excellent electrochemical properties can be credited to the singular nano urchin-like structure and a series of redox reactions, which not only involve Co^2+^ and Mn^2+^, but also come from Ni^2+^, thus increasing the redox peak. The specific redox reaction mechanism is as follows:

NiMoO_4_: NiMoO_4_ → Ni^2+^ + MoO_4_^2−^ (4)

Ni^2+^ + 2OH^−^ → Ni(OH)_2_ (5)

Ni(OH)_2_ + OH^−^ → NiOOH + H_2_O + e^−^ (6)

MnCo_2_O_4_: MnCo_2_O_4_ + H_2_O + OH^−^ → MnOOH + 2CoOOH + e^−^ (7)

MnOOH + OH^−^ → MnO_2_ + H_2_O + e^−^ (8)

CoOOH + OH^−^ → CoO_2_ + H_2_O + e^−^ (9)

Figure 5d shows the GCD curves of NiMoO_4_, MnCo_2_O_4_ and NiMoO_4_@MnCo_2_O_4_ composite electrode material measured at the current density of 1 mA/cm^2^. It is observed that the charge and discharge time of NiMoO_4_@MnCo_2_O_4_ composite electrode material is the longest, which corresponds to the maximum CV curve area of NiMoO_4_@MnCo_2_O_4_ in Figure 5c. By calculation, the specific capacitances of the three electrodes can reach 1656, 224 and 3000 mF/cm^2^. The specific capacitance of NiMoO_4_@MnCo_2_O_4_ is compared, as shown in Table 1, which is higher than that of some previous literatures [32,33,34,35,36]. The charging–discharging time of NiMoO_4_@MnCo_2_O_4_ composite electrode material is the longest, and the symmetry of the charging and discharging cycle indicates that the electrode has excellent reversibility. The capacitance performance is attributed to the nano urchin-like morphology of the material, which provides a larger electrolyte contact area. Therefore, the electrochemical properties of composite electrode material are improved. To further explore the charge transfer ability of the prepared electrodes, EIS measurements were carried out, as shown in Figure 5e. The inset exhibits that compared with two single electrodes, in the high frequency region, the NiMoO_4_@MnCo_2_O_4_ sample has a smaller semicircle arc and x-axis intercept, which represents the charge transfer resistance (R_ct_) and solution resistance (R_s_), indicating that the composite has a faster ion-electron transfer rate at the electrode and electrolyte interface, and smaller intrinsic resistance. The corresponding R_s_ values of NiMoO_4_, MnCo_2_O_4_, and NiMoO_4_@MnCo_2_O_4_ are 0.91, 0.77 and 0.67 Ω, respectively. In the low frequency region, the composite material shows the higher straight-line slope, which accounts for faster electrolyte ion mobility. Cycling performance (10 mA cm^−2^) of the as-prepared electrodes is displayed in Figure 5f. Compared with NiMoO_4_ (75%) and MnCo_2_O_4_ (45%), NiMoO_4_@MnCo_2_O_4_ (96%) shows a better cycling lifespan after undergoing the charging–discharging process 10,000 times.

**Figure 6:**
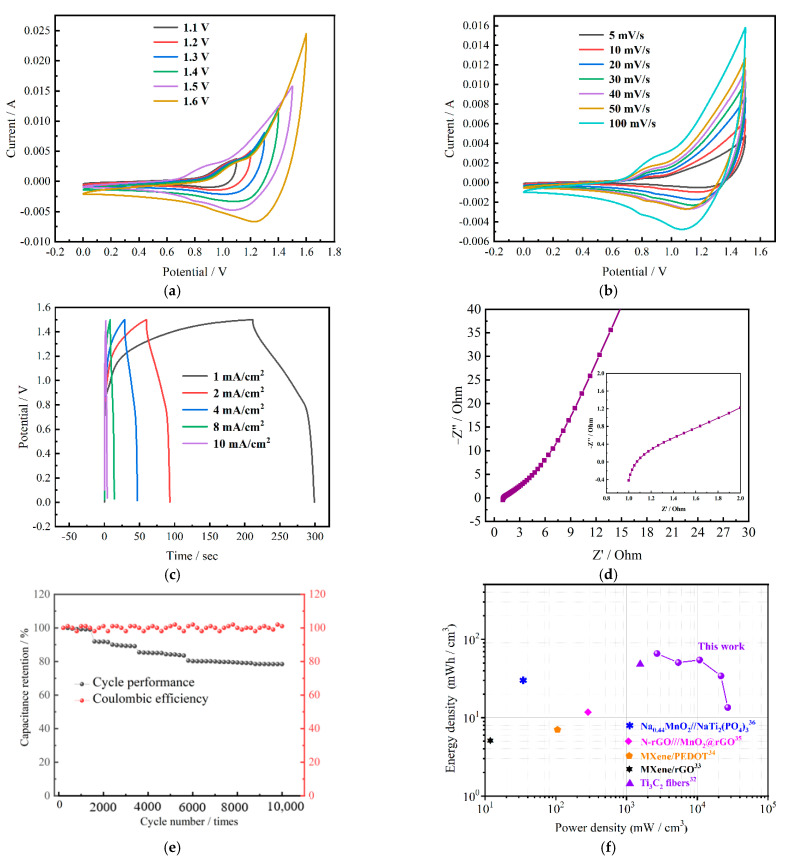
Electrochemical testing of NiMoO_4_@MnCo_2_O_4_ composite assembled devices: (**a**) Cyclic voltammetry curves under different voltage windows; (**b**) Cyclic voltammetry curves of different scanning speeds; (**c**) GCD curves with different current densities; (**d**) Impedance diagram (inset is the high-frequency region); (**e**) Cycling stability and coulombic efficiency; (**f**) Ragone plots.

In order to study the application of NiMoO_4_@MnCo_2_O_4_ in SCs, the positive electrode and negative electrode of ASCs are NiMoO_4_@MnCo_2_O_4_ electrode and active carbon (AC) electrode, respectively. Figure 6 shows the electrochemical curves of the assembled device. Figure 5a shows the CV curves at the scanning rate of 100 mV/s. The voltage windows of the device are 1.1 V, 1.2 V, 1.3 V, 1.4 V, 1.5 V and 1.6 V, respectively. The shapes of all curves are nearly the same, indicating that the device can operate at 1.1 V–1.6 V and the maximum voltage window can reach 1.6 V at the same time. Figure 6b shows the CV curves of NiMoO_4_@MnCo_2_O_4_//AC at scanning rates of 5–100 mV/s. With the increase in scanning rate, the shapes of the CV curves increase, which is mainly attributed to the synergy between materials. These curves have obvious redox peaks, indicating that the asymmetric SCs have pseudocapacitance characteristics. Meanwhile, with increasing scanning rate, the integral area of the curves is enhanced. The GCD curves with different current densities are shown in Figure 6c, which indicates that the linear trend of the curve is obvious at high current densities. The voltage window is 1.5 V, and the surface capacitance of the device can be calculated according to the formula. When the current densities are 1, 2, 4, 8 and 10 mA/cm^2^, the surface capacitances are 58.53, 22.73, 12.13, 1.9 and 1.13 mF/cm^2^, respectively. Figure 6d shows the charge transfer characteristics of the prepared electrode studied by EIS test. The slope is larger in the low frequency region, indicating that the diffusion resistance of the assembled asymmetric SC is lower. The inset shows the R_s_ value is only 1 Ω. Figure 6e shows the long cycling test with 10,000 times at 10 mA cm^−2^ and coulombic efficiency. The capacity retention rate of the assembled asymmetric SC is 78.4%. The decrease in capacity may be due to the morphology damage caused by long-term redox reaction of electrode materials, which reduces the potential activity of the surface of the material. The coulombic efficiency of ASCs keeps nearly 100% during 10,000 charging–discharging tests. From Figure 6f, the Ragone plot offers an expression of the trend of the energy density with the corresponding power density. Importantly, the maximum energy density of the NiMoO_4_@MnCo_2_O_4_//AC device reaches 90.89 mWh/cm^3^ at the power density of 3726.7 mW/cm^3^, which is better than some reported devices [37,38,39,40,41].

## 4. Conclusions

A new type of NiMoO_4_@MnCo_2_O_4_ composite electrode material has been successfully prepared on nickel foam by the two-step hydrothermal method, and its phase structures, micromorphology and electrochemical properties are characterized and analyzed. Due to the synergistic effect between the NiMoO_4_ nano pompon-like structure and MnCo_2_O_4_ nano needle-like structure, the prepared nano urchin-like NiMoO_4_@MnCo_2_O_4_ core–shell nanostructure presents good pseudocapacitance properties. NiMoO_4_@MnCo_2_O_4_ samples show better electrochemical performance than single NiMoO_4_ or MnCo_2_O_4_ electrode materials, which exhibit a high specific capacitance of 3000 mF/cm^2^. After 10,000 cycles, the capacity retention rate is 96%. In addition, the NiMoO_4_@MnCo_2_O_4_//AC assembled device delivers a high energy density of 90.89 mWh/cm^3^ at a power density of 3726.7 mW/cm^3^.

## Figures and Tables

**Table 1:** Electrochemical performance comparison of NiMoO_4_@MnCo_2_O_4_ with previous literatures.

Materials	Capacity	Current Density	Electrolyte	Capacitance Retention	Ref.
NiCo_2_O_4_/rGO/NiO	2.644 F cm^−2^	1 mA cm^−2^	3 M KOH	97.5% (3000 cycles)	[32]
Fe_2_O_3_/Fe dendrite	2.166 F cm^−2^	1 mA cm^−2^	1 M KOH	90% (1000 cycles)	[33]
NiCo_2_O_4_/C	2.057 F cm^−2^	1 mA cm^−2^	2 M KOH	81% (10,000 cycles)	[34]
rGO/PPy	0.807 F cm^−2^	1 mA cm^−2^	1 M H_2_SO_4_	78% (2000 cycles)	[35]
C@MnNiCo-OH/Ni_3_S_2_	2.332 F cm^−2^	1 mA cm^−2^	3 M KOH	89.45% (5000 cycles)	[36]
NiMoO_4_@MnCo_2_O_4_	3 F cm^−2^	1 mA cm^−2^	3 M KOH	96% (10,000 cycles)	This work
